# VP1–141 is a determinant of a Vero cell-adapted Coxsackievirus A10 for vaccine development

**DOI:** 10.1371/journal.pntd.0014396

**Published:** 2026-06-02

**Authors:** Chi-Hsun Chen, Chih-Yeu Fang, Yu-Sheng Shen, Jen-Ren Wang, Suh-Chin Wu, Chia-Chyi Liu

**Affiliations:** 1 Institute of Biotechnology, National Tsing Hua University, Hsinchu, Taiwan; 2 National Institute of Infectious Diseases and Vaccinology, National Health Research Institutes, Zhunan, Miaoli County, Taiwan; 3 National Institute of Infectious Diseases and Vaccinology, National Health Research Institutes, Tainan, Taiwan; 4 Department of Medical Laboratory Science and Biotechnology, National Cheng Kung University, Tainan, Taiwan; 5 International Master Program of Translation Medicine, College of Engineering and Science, National United University, Miaoli, Taiwan; The University of the West Indies, JAMAICA

## Abstract

Coxsackievirus A10 (CVA10) infection primarily causes hand, foot, and mouth diseases (HFMD) in children. As CVA10 is one of the most widespread enteroviruses, the development of a CVA10 vaccine is essential. It could also be used to create a multivalent vaccine for preventing HFMD alongside other enteroviruses. Most clinical isolates of CVA10 are difficult to propagate in Vero cells, while only a few pre-screened CVA10 clones could. In this study, we have successfully isolated a highly Vero cell-adapted CVA10 strain (CVA10-V) from the parental CVA10. After propagation of CVA10-V in Vero cells, the purified full-particle (F-particle) of CVA10-V could induce strong immunogenicity in mice model, while the empty-particle of CVA10-V did not. The CVA10-V F-particle were mixed with EV-A71 bulk for immunization and a good neutralization response to each virus were observed. In this study, a RD-propagated CVA10 strain (CVA10-R) was not unable to infect Vero cells. Five amino acid variations of P1 protein (470, 664, 705, 792, and 804) were noted between the CVA10-V and CVA10-R. Infectious clones with single-site mutation representing these five locations were constructed to investigate the effects of these variations on Vero infectivity. Substitution of D from E at position VP1–141 (P1-705) was found to be the critical determinant for CVA10-V infection of Vero cells. Variation at this position was found to affect CVA10’s ability to attach to Vero cells. Our study pinpoints the critical sites of viral P1 protein that render the Vero-adaptation and this Vero-adapted CVA10-V strain would be beneficial for multivalent HFMD vaccine development using the Vero cell culture system.

## Author summary

Coxsackievirus A10 (CVA10) is primarily associated with Hand, foot, and mouth diseases (HFMD), which can occasionally lead to severe complications or death in children. The current bottleneck in the CVA10 vaccine development is that only few pre-screened CVA10 strains can proliferate in Vero cell, which is the cell line commonly used for enterovirus and coxsackievirus propagation. Determining the variations in the viral structural proteins that lead to virus infection in Vero cells is important for understanding the mechanisms of CVA10 cell tropism. Also, a Vero-adapted CVA10 is useful for large-scale production of a potent CVA10 vaccine. Here, we screened and obtained two CVA10 strains (CVA10-R/CVA10-V), which expressed distinct growth characteristics in Vero and RD cells. Five amino acid variations in the P1 transcript of the two viruses were determined. Our study has identified that VP1–141 (P1-705) is a critical amino acid site for CVA10-V infection in Vero cells, and this CVA10-V strain could be used as a suitable vaccine strain in the Vero cell culture system.

## Introduction

Coxsackievirus A10 (CVA10) is one of enteroviruses that causes hand, foot, and mouth diseases (HFMD), with symptoms including fever, sore throat, erythematous, and onychomadesis, particularly in children [1]. In recent years, epidemiological surveys have shown that there have been several outbreaks of CVA10-related HFMD worldwide, and this disease is a serious public health issue for children [2–11]. Several CVA10 vaccine candidates have been developed, including inactivated whole-virion and virus-like particle (VLP) vaccines [3,12–15]. To provide a broad protection against HFMD, it is essential to combine CVA10 with other enteroviruses in order to create a multivalent vaccine for the prevention of HFMD.

CVA10, a non-enveloped RNA virus with a genome size of approximately 7.4 Kbp, is classified in the family Picornaviridae [16–18]. KREMEN1 is reported to be a major cellular receptor for CVA10 and several other coxsackieviruses (CVA2, CVA3, CVA4, CVA5, CVA6, CVA12), but not for CVA7, CVA14, CVA16 and EV-A71 [19–22]. Currently, whole virions required for Poliovirus and Enterovirus A71 (EV-A71) vaccine productions are mainly harvested from the Vero cell cultures, which is a standard platform for enteroviral vaccine production [14]. However, it has been reported that most CVA10 clinical isolates have low proliferation efficiency in Vero cells, while only a few pre-screened, Vero cell-adapted CVA10 strains could propagate in Vero cells [11–13,20,23–25]. To overcome this issue and increase the yield of CVA10 virus, a serum-free HEK293A suspension culture system has been developed for large-scale virion production of CVA10 [26]. Nevertheless, developing a highly Vero cell-adapted CVA10 strain is important for vaccine production, given that the Vero cell-based vaccine platform is the gold standard for the pharmaceutical production of enteroviral vaccines.

RNA viruses exhibit a high mutation rate due to the lack of proofreading mechanism and the viral RNA polymerase is error-prone, which could generate many quasispecies of RNA viruses [27]. Several studies have pointed out that serial passages of isolated CVA10 in cell culture have resulted in accumulated mutations of CVA10 that could affect the viral growth characteristics in cells [11,13,20,23–25]. Some Vero-adapted CVA10 strains have been reported to exhibit sequence variations in viral structural proteins, but the critical sites that lead to such adaptation remain undetermined.

In this study, we developed a Vero cell-adapted CVA10 strain and characterized its adaptation characteristics. By amplifying the diversity of CVA10 pool before screening, we obtained a host-adapted CVA10 strain in Vero cells. Based on the variations of viral protein (VP) sequences, we reported and defined the critical changes of the CVA10 for its adaptation to Vero cells. The P1-705 residue was found to be involved in virus attachment to Vero cells. Our study provides an insight into the evolvement of enteroviral tropism to Vero cells. Most importantly, a feasible CVA10 vaccine candidate could be developed from the Vero cell-based vaccine production system and used in combination with the EV-A71 vaccine to create a bivalent HFMD vaccine.

## Materials and methods

### Ethics statement

Animal protocols have been reviewed and approved by the Animal Care and Use Committee of the National Health Research Institutes (Protocol number: NHRI-IACUC-110008-A and NHRI-IACUC-111114-A).

### Cells, media, and viruses

Vero, RD, and HEK293A cells were cultured in Dulbecco’s modified Eagle’s medium (DMEM, Gibco) with 10% fetal bovine serum (FBS) for virus cultivation. The CVA10-M2014 strain was obtained from National Cheng Kung University Hospital, Taiwan. The EV-A71 E59 strain was obtained from the Centers for Disease Control, Taiwan. For virus propagation and purification, Vero cells were cultured in serum-free medium VP-SFM (Gibco). More details were described in S1 File.

### The adaption and isolation of CVA10 strains

**Fig 1:**
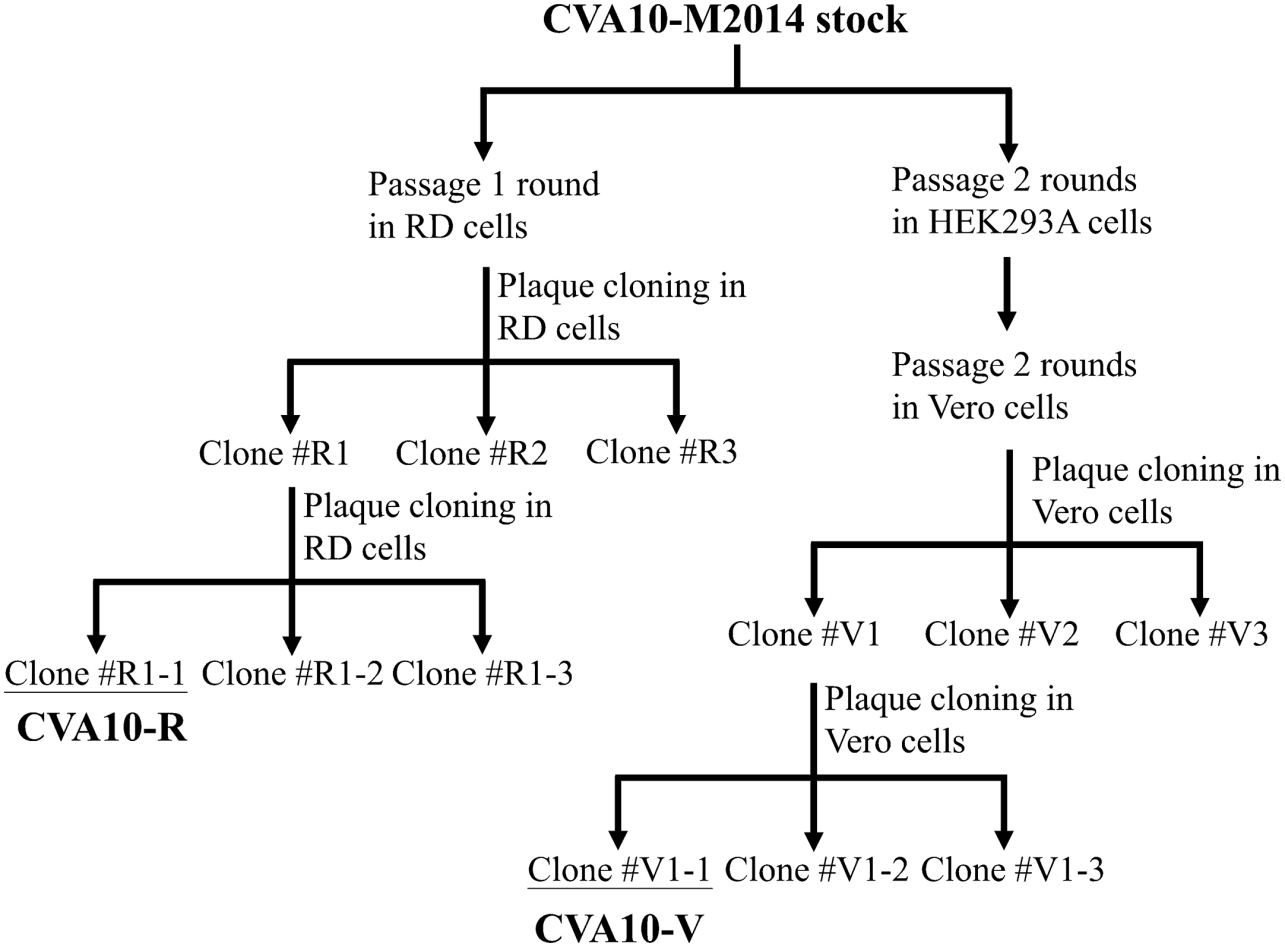
Host adaptation of CVA10-M2014 strain. The parental CVA10-M2014 were subjected to passaging in different cells and later independent virus strains were isolated by plaque cloning. CVA10-R originated from CVA10-M2014 passaging in RD cells, and CVA10-V originated from passaging CVA10-M2014 in HEK293A cells for 2 rounds, then passaging in Vero cells for 2 rounds.

The primary CVA10-M2014 strain was passaged twice in HEK293A cells to propagate the virus yields, which is then used to infect Vero cells. After two screening passages in Vero cells, the adapted CVA10 strains were isolated by plaque cloning (1.1%, low-melting agarose with fresh medium). In the first-round of plaque cloning selection, three plaques were selected and amplified by Vero cell culture. The #V1 clone was subjected to the second-round plaque cloning and three plaques were selected. Finally, the #V1-1 clone was amplified hereafter and referred to as the CVA10-V strain (Fig 1). The same primary stock was also passaged once with RD cells, independently. During two rounds of plaque cloning in RD cells, the #R1-1 clone was selected and referred to as the CVA10-R strain (Fig 1). The growth profile of CVA10-V and CVA10-R was evaluated by infecting Vero and RD cells.

### Determination of the virus titers and plaque assay

The virus titers were determined by the median tissue culture infectious dose (TCID_50_) assay. Serially diluted virus samples (from 10^-1^ to 10^-8^) were added to RD cells in 96-well plates, and 6 replicate samples were used for each dilution. The 96-well plates were incubated for 6 days at 37 °C, and the TCID_50_ values were measured by counting the cytopathic effects (CPE) on infected RD cells. The TCID_50_ values were calculated using the Reed–Muench method. The viral morphology was observed by plaque assay that was measured in duplicate after adding 100-μL samples of ten-fold, serially diluted virus samples to monolayer RD cells in six-well plates. After 1-hour incubation at 37 °C, 4 mL of medium containing 10% FBS and 1.1% methylcellulose (4 mL/well) was added to each well. After incubation for three days, the wells were then washed and fixed with a 3.7% formaldehyde solution. After fixation, the wells were then stained with the crystal-violet-staining (0.1 M citric acid containing 0.1% w/v crystal violet).

### The viral particles of CVA10-V preparation and immunogenicity studies

The propagation, purification, and characterization of CVA10-V particles were evaluated as reported in our previous study [28]. The viral proteins were detected by SDS-PAGE and western blotting analysis. The anti-CVA6 VP1 rabbit polyclonal antibody GTX132346 (GeneTex) was used to recognize the CVA10 antigen [26]. The CVA10 empty (E)-particle and full (F)-particle were purified by ultracentrifugation and observed to transmission electron microscopy (TEM) as described in our previous report [29]. The purified samples of CVA10-V particles were inactivated using formalin solution (v/v 1:4,000 dilution) [26]. As described in our previous study, a group of 6 female BALB/c mice (6–8 weeks old) were immunized intramuscularly (i.m.) with AlPO_4_-formulated viral antigens (viral proteins 0.5 µg with AlPO_4_ 60 µg in 0.2 mL) [26]. Formalin-inactivated EV-A71 bulk (sample-05), which was evaluated in our previous study [30], was mixed and used in the bivalent study. For bivalent test, EV-A71 bulk (0.5 μg) and CVA10-V F-particle (0.5 μg) were mixed with 60 µg AlPO_4_ in 0.2 mL. Mice were boosted once with identical content at two-week intervals after priming. Blood from the immunized mice was collected one week after the final boost, and the serum was used for virus neutralization study. The neutralizing antibody titer (Nt titer) was determined as described previously [26]. More details were described in S1 File.

### Viral genome sequence analyses

Viral RNA was extracted from 5 x 10^6^ TCID_50_ of CVA10 strains using RNAzol reagent (Molecular Research). Reverse transcription was performed using oligo dT primers with reverse transcriptase ((Thermo) to obtain the viral cDNA. The viral cDNA was amplified by PCR with specific primers (S1 Table) which covering the entire genome of CVA10. The PCR products were then sequenced.

### Construction of the full-length CVA10-R and -V cDNA clones and mutants

The cDNA-derived CVA10 infectious clones were constructed with DNA fragments covering the genomic sequences of CVA10-R and CVA10-V strains. The full-length CVA10-R cDNA clone (CVA10-Rc) and the full-length CVA10-V cDNA clone (CVA10-Vc) comprising 7.4 K nucleotides (nt) has been generated by PCR, and these PCR products were then digested with restriction enzymes at both ends and inserted into the pCMV-HA plasmid (Clontech) as described previously [31]. Specific primers were used to construct the site-specific mutations of infectious clones for functional analysis (S2 Table).

### Recovery of CVA10 cDNA clone virus

For CVA10 virus recovery, RD cells were transfected with the cDNA infectious clone plasmid (2 μg) with 8 μL of T-Pro NTR II transfection reagent (T-Pro Biotechnology) in 6-well plates. The culture supernatants (P1 passage) were collected after incubation for 3–6 DPI. The P1 viruses were subsequently propagated in RD cells (P2 passage) for subsequent experiments.

### Virus attachment assay

To understand how variations in viral structural proteins affect virus attachment to Vero cells, a virus attachment assay was performed as previously described [32]. Vero cells were seeded in 12 well plates and incubated at 37°C overnight. Then, plates containing confluent Vero cells were pre-chilled at 4°C for 1 hour. Testing viruses were diluted at the multiplicity of infection (MOI) = 0.001, and added to the chilled cells on ice. The cells were then incubated at 4°C for 2 hours. Supernatant was then removed from the cells, and cells were washed gently with 2 mL of ice-cold PBS twice. After washing, 500 μL of NucleoZOL (Macherey-Nagel) was added to the cells for total RNA isolation. The total RNA was then used to synthesize cDNA by a RevertAid H Minus First Strand cDNA Synthesis Kit (Thermo Scientific). The synthesized cDNA was then mixed with HOT FIREPol EvaGreen qPCR Mix Plus (Solis BioDyne) and the recommended qPCR cycling protocol was followed for qPCR analysis. The cycling conditions for this reaction were one cycle at 95 °C for 12 min; 40 cycles at 95 °C for 15 sec, 60 °C for 20 sec, 72 °C for 20 sec. qPCR cycling and analysis was performed on QuantStudio 6 Flex (Applied Biosystems). Specific primers were used for qPCR analysis (S3 Table).

### Statistical analysis

A one-way ANOVA test was used to analyze the results. An unpaired Student’s t test was used to analyze the results. The results were considered to be statistically significant when p < 0.05. The symbols *, ** and *** were used to indicate p < 0.05, p < 0.01, and p < 0.001, respectively. All experiments of virus growth curves were done in duplicates.

## Results

### Host-adapted CVA10 strains isolation

To increase the strain diversity of CVA10-M2014, the CVA10 seed were propagated in HEK293A cells for two passages. The resulting CVA10 was used to infect Vero cells and CPE was observed. During the two screening passages in Vero cells, the propagated CVA10 clones were isolated by plaque cloning with Vero cells for each passage, and a single CVA10 clone from the secondary cloning (#V1-1) was selected as the “CVA10-V” strain (Fig 1). In contrast, the same CVA10 seed were propagated in RD cells for one passage. The RD-propagated CVA10 clones were also isolated by plaque cloning with RD cells in the following two passages, and a single CVA10 clone of the secondary cloning (#R1-1) was selected as the “CVA10-R” strain (Fig 1).

### Characterization of the Vero-adapted CVA10 strains

**Fig 2:**
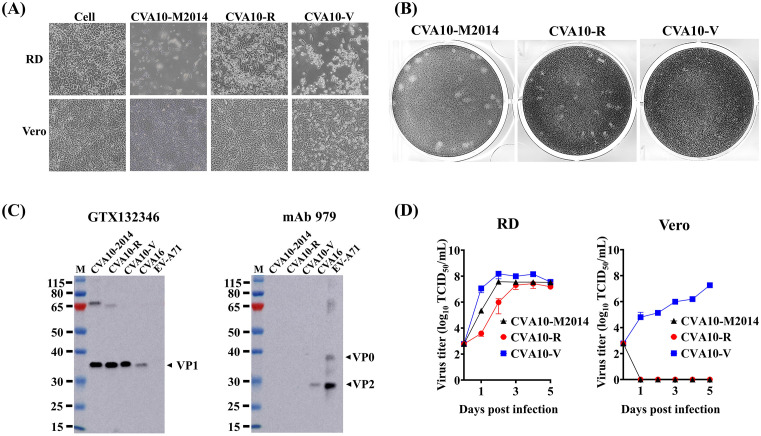
Characterization of the CVA10-R and CVA10-V in RD and Vero cell lines. **(A)** The cytopathic effects of CVA10-M2014, CVA10-R and CVA10-V in RD and Vero cells. RD cells and Vero cells were infected with CVA10-M2014, CVA10-R and CVA10-V at MOI = 0.001 respectively. The cells were observed after 3 days for cytopathic effects. **(B)** The plaque phenotype of CVA10-M2014, CVA10-R and CVA10-V in RD cells. **(C)** Western blot analyses of the CVA10-R and CVA10-V using the GTX132346 and mAb979 antibody. Each sample was loaded with 2 x 10^6^ TCID_50_/mL virus titers for western blot analyses. Lane 1: CVA10-M2014; Lane 2: CVA10-R; Lane 3: CVA10-V; Lane 4: CVA16 N5079; and Lane 5: EV-A71 E59. **(D)** The growth kinetics of CVA10-M2014, CVA10-R and CVA10-V in RD and Vero cell lines. Viruses were seeded at MOI = 0.001 and incubated at 37 °C for 5 days. Virus samples were collected daily and virus titers were calculated via TCID_50_ assay. The experimental data is presented as the mean ± standard deviation (SD) of duplicates.

The parental CVA10-M2014, CVA10-R and CVA10-V were used to infect both RD and Vero cells at MOI = 0.001. After 3 days, CPE was observed in CVA10-M2014, CVA10-R and CVA10-V infected RD cells. In Vero cells, only the CVA10-V induced CPE, while CVA10-R and CVA10-M2014 did not. (Fig 2A). In RD cells, plaques of CVA10-R and CVA10-M2014 were distinct and clear, while plaques of CVA10-V were small and vague (Fig 2B). Due to the lack of a CVA10-specific antibody, a polyclonal anti-CVA6 antibody (GTX132346) that has cross-reactivity to CVA10 was used to detect CVA10 antigens in this study [26]. Monoclonal antibody mAb979, which can detect EV-A71 and CVA16, but not CVA10, was used as a negative control. CVA10 were seeded at a MOI = 0.001 in RD cells and incubated at 37 °C until CPE. Cell lysate containing CVA10 were then collected and subjected to Western blotting. The result showed that CVA10-M2014, CVA10-R and CVA10-V proteins were detected by the GTX132346 antibody but not by mAb979 (Fig 2C). CVA10-M2014, CVA10-R and CVA10-V could easily proliferate in RD cells. The virus titer of CVA10-V reached 10^7^ TCID_50_/mL after 5 DPI in Vero cells, but no CVA10-R virus or CVA10-M2014 virus could be detected in Vero cell culture (Fig 2D). These results show that CVA10-R and CVA10-V present different cell tropism for RD and Vero cells.

In order to examine the gene stability of Vero-adapted CVA10-V, we serial passaged CVA10-V in Vero cells for 6 generations and sequenced P6 viruses and aligned their nucleotide and amino acids with the original virus and showed the differences in S4 Table. Sequencing results show that after 6 generations of passaging in Vero cells, two nucleotide mutations were detected in the CVA10-V-P6 virus genome, resulting in two amino acid mutations. We also measured the titers of the passaged viruses in the following figure (S1 Fig). The results show that CVA10-V viruses retained infectivity in Vero cells throughout 6 generations of passaging.

### Purification and immunogenicity of CVA10-V particles

**Fig 3:**
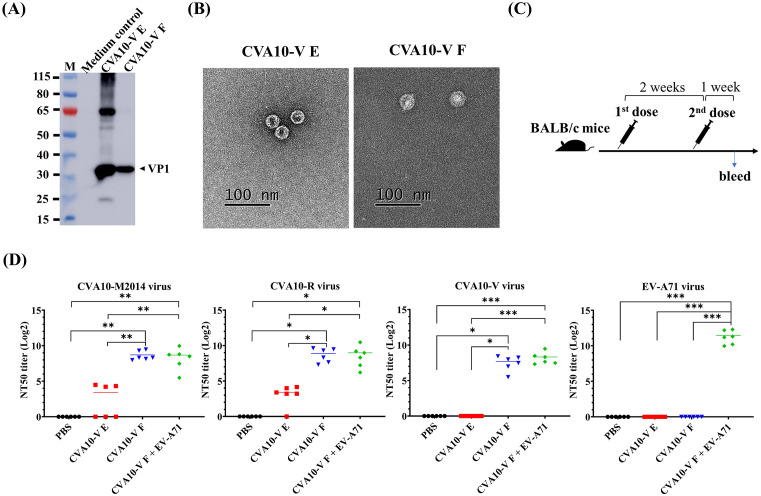
The neutralization titers of antisera in mice against CVA10-R, CVA10-V and EV-A71. **(A)** CVA10-V E-particle (CVA10-V E) and CVA10-V F-particle (CVA10-V F) were analyzed by Western blot using GTX132346 antibody. **(B)** Transmission electron microscopy of CVA10-V E-particle and CVA10-V F-particle. **(C)** The flow chart of immunization. 6 ~ 8 weeks old female BALB/c mice were immunized via the intramuscular route (i.m) with formalin inactivated CVA10-V E-particle, CVA10-V F-particle, CVA10-V F-particle mixed with EV-A71 vaccine respectively. Two doses were administrated, and blood was collected one week after the final dose. **(D)** Neutralization titers of mice. Sera of mice was used to perform neutralization assay against CVA10-M2014, CVA10-R, CVA10-V and EV-A71 viruses respectively. The experimental data is presented as the mean ± standard deviation (SD) of six replicates (N = 6).

Previous studies have shown that the E- and F-particles of enteroviruses may exhibit different immune characteristics [29]. To investigate the immunogenicity of CVA10-V E- and F-particles produced from cell culture, the CVA10-V were propagated using serum-free Vero cell roller bottle culture, and two particles were purified by sucrose gradient zonal ultracentrifugation as described previously [26,29]. Four groups of mice were immunized with control or formalin-inactivated antigens (PBS only, E-particle, F-particle of CVA10, and CVA10 F-particle mixed with EV-A71 bulk) as indicated in Section 2.4. The results show that the CVA10-V F-particle could induce good neutralization antibody responses against CVA10-M2014, CVA10-V and CVA10-R, but the CVA10-V E-particle failed to do so (NT < 16) (Fig 3). The CVA10-V F-particle mixed with EV-A71 bulk induced good neutralization antibody responses against both EV-A71 and CVA10 (Fig 3). Neutralization antibody responses were also tested against CVA6 and CVA16, but all groups failed to cross neutralize these viruses (S2 Fig). These results indicate that CVA10-V F-particle could be used with EV-A71 as a bivalent CVA10/EV-A71 vaccine candidate.

### Construction of CVA10-R and CVA10-V infectious clones

CVA10-R and CVA10-V stocks were collected and the viral RNAs were extracted for reverse-transcription to cDNA for cloning and sequencing. The full genomes of CVA10-R and CVA10-V were sequenced, and the differences in sequence of P1 gene were compared and listed in Table 1.

**Table 1:** The P1 genome sequence comparison of CVA10-V, CVA10-R, parental stain.

Nucleotide change in polyprotein	Amino acid positions	VP protein (position)	CVA10-M2014 (parental stain)	CVA10-R strain	CVA10-V strain	References of Vero-adapted CVA10 strains reported with identical mutation sites with CVA10-V
a1408g	470	VP3 (146)	T	T	A	**ND**
a1990g	664	VP1 (100)	T	T	A	**V6-19/XY/CHN/2017** [11] **YNKG1-7** [11]
g2115t	705	VP1 (141)	E	E	D	**S0273b** [23]
g2374a	792	VP1 (228)	M	V	M	**V6-19/XY/CHN/2017** [11] **YNKG1-7** [11] **CVA10/SD/CHN/09** [33] **Kowalik** [23] **S0148b** [23] **S0273b** [23] **CVA10-AJK93551** [20]
g2411a	804	VP1 (240)	E	R	K	**CVA10/SD/CHN/09** [33] **S0273b** [23]

**Fig 4:**
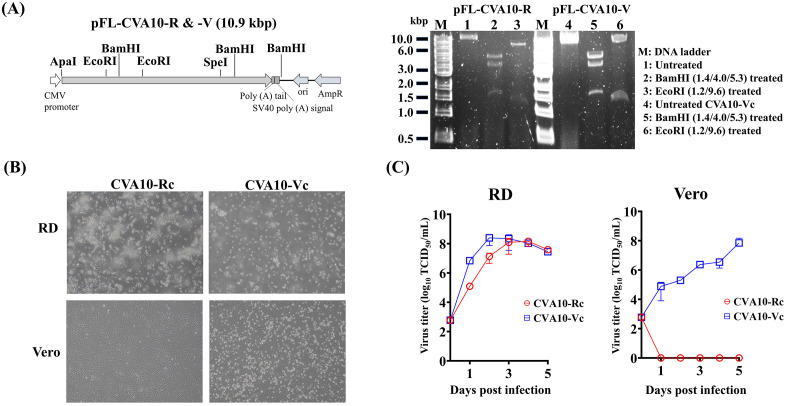
The construction of the infectious cDNA-derived CVA10 clones. The pFL-CVA10 plasmid contains a full-length CVA10-V or CVA10-R cDNA inserted into the modified pCMV-HA vector using restriction sites. **(A)** The pFL-CVA10 plasmids is 10.9 kb in length (lane 1) and contained three Bam HI (lane 2), and two Eco RI (lane 3) restriction enzyme sites. **(B)** The CPE of CVA10-Vc and CVA10-Rc infected RD and Vero cells. **(C)** The growth kinetics of the CVA10-Vc and CVA10-Rc in RD and Vero cells at MOI = 0.001. Virus samples were collected daily and virus titers were calculated via TCID_50_ assay. The experimental data is presented as the mean ± standard deviation (SD) of duplicates.

CVA10-R strain was identified as having almost the same sequence as the parental CVA10 stain, except for the amino acid 792 position, which is valine (V) instead of methionine (M). The CVA10-V strain differs from the CVA10-R strain by five nucleotides, resulting in five amino acid differences (Table 1). CVA10-R and CVA10-V infectious clones were constructed by PCR cloning into a pCMV-HA plasmid (primers list: S1 Table), and the restriction profiles of CVA10 infectious clones were identified using specific enzyme digestion (Fig 4A). These cDNA clones were transfected into RD cells, and the amount of cDNA-derived CVA10-R (CVA10-Rc) and cDNA-derived CVA10-V (CVA10-Vc) were determined by TCID_50_. The collected viruses were used to infect RD and Vero cells. After 3 days, both CVA10-Rc and CVA10-Vc could induced CPE in RD cells. In contrast, CVA10-Vc could induce CPE in Vero cells while CVA10-Rc could not (Fig 4B). The virus replication kinetics of CVA10-Rc and CVA10-Vc were evaluated in RD and Vero cells. As in previous experiments of plaque-isolated CVA10-R and CVA10-V (Fig 2D), the CVA10-Vc virus titer reached 10^7^ TCID_50_/mL while the CVA10-Rc was undetectable in Vero cells (Fig 4C). These results confirmed that the properties of CVA10-Rc and CVA10-Vc infectious clones were identical to those of plaque-isolated CVA10-R and CVA10-V.

### Determine the crucial amino acid changes for Vero cell adaptation in CVA10 clones

**Fig 5:**
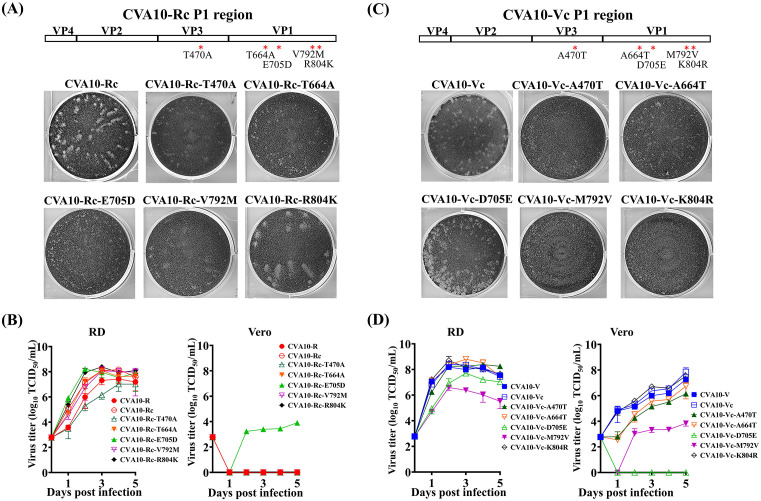
The construction and growth profiles of the infectious CVA10-Rc and CVA10-Vc mutants. **(A)** The construction of the infectious CVA10-Rc mutants and plaques. **(B)** The growth profiles of the infectious CVA10-Rc mutants. RD and Vero cells were infected with passage 3 virus at MOI = 0.001. Virus samples were collected daily and virus titers were calculated via TCID_50_ assay. The experimental data is presented as the mean ± standard deviation (SD) of duplicates. **(C)** The construction of the infectious CVA10-Vc mutants and plaques. **(D)** The growth profiles of the infectious CVA10-Vc mutants. RD and Vero cells were infected with passage 3 virus at MOI = 0.001. Virus samples were collected daily and virus titers were calculated via TCID_50_ assay. The experimental data is presented as the mean ± standard deviation (SD) of duplicates.

Site-direct mutagenesis was used for construction of single-mutation clones of CVA10-R and CVA10-V. The CVA10-Rc and CVA10-Vc plasmids were used as templates, and specific primers were designed for site-direct PCR (S2 Table). Five single amino acid substitution CVA10-Rc clones (CVA10-Rc-T470A, CVA10-Rc-T664A, CVA10-Rc-E705D, CVA10-Rc-V792M, and CVA10-Rc-R804K) were constructed and recovered in RD cells. The morphology of plaques formed by these infectious clones presented different sizes and shape compared to CVA10-Rc (Fig 5A). The growth kinetics of these CVA10-Rc single-substitution viruses were evaluated in RD and Vero cells. All infectious clones could grow in RD cells, but only the CVA10-Rc-E705D virus could propagate in Vero cells (Fig 5B). Additionally, five single-substitution clones of CVA10-Vc (CVA10-Vc-A470T, CVA10-Vc-A664T, CVA10-Vc-D705E, CVA10-Vc-M792V, and CVA10-Vc-K804R) were constructed and recovered in RD cells. The plaques morphology of these infectious clones presented different sizes compared to CVA10-Vc (Fig 5C). Plaques of the infectious clones were then measured and categorized into three different size categories (S5 Table). The growth kinetics of these single-substitution CVA10-Vc viruses were evaluated in RD and Vero cells. All infectious clones could grow in RD cells, but only the CVA10-Vc-D705E virus could not propagate in Vero cells (Fig 5D). These results indicate that the amino acid position P1-705 is a critical site and the change from E to D in this position confers CVA10 the ability to propagate in Vero cells.

### The cell attachment ability of CVA10 P1-705 mutants

**Fig 6:**
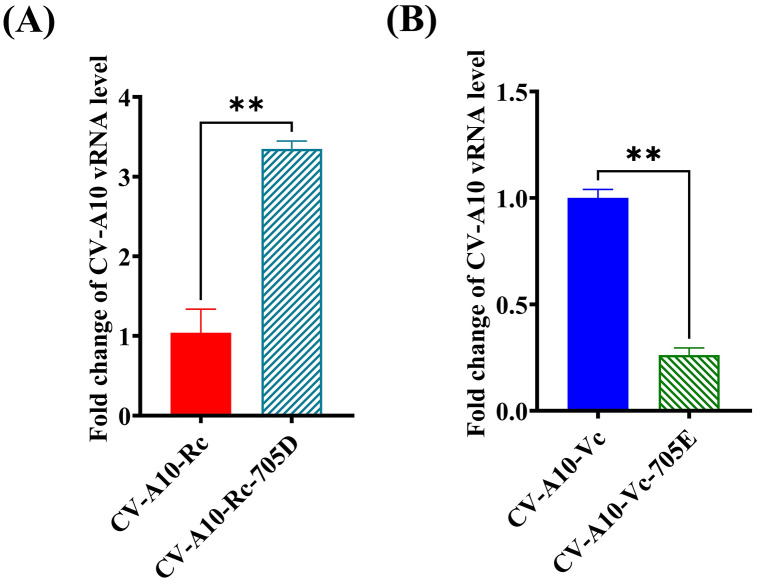
The virus attachment assay of the CVA10-Rc and CVA10-Vc mutants. CVA10-Rc, CVA10-Rc-E705D, CVA10-Vc and CVA10-Vc-D705E were subjected to virus attachment assay as described in Material and Methods. **(A)** The relative viral RNA content of CVA10-Rc-E705D virus was compare with that of CVA10-Rc virus by qRT-PCR. The fold change in viral attachment was a 3.21-fold increase (p = 0.0089). **(B)** The relative viral RNA content of CVA10-Vc-D705E virus was compare with that of CVA10-Vc virus by qRT-PCR. The fold change in viral attachment was a 3.83-fold decrease (p = 0.0025). The experimental data is presented as the mean ± standard deviation (SD) of duplicates.

To understand whether the amino acid residue at the P1-705 position affects the virus’s ability to attach to Vero cells, the CVA10-Rc-E705D and CVA10-Vc-D705E mutants were evaluated for their binding ability in an attachment assay. Our results showed that, by changing the residue from E to D at P1-705, the attachment ability of the CVA10-Rc-E705D was 3.21-fold higher than that of the CVA10-Rc. In contrast, when the P1-705 residue of CVA10-Vc was changed from D to E, the attachment ability of the CVA10-Vc-D705E was 3.83-fold lower than that of the CVA10-Vc (Fig 6). These results suggest that the P1-705 site plays a vital role in the attachment of CVA10 to Vero cells, which could be a critical factor determining the infectivity of CVA10 in Vero cells.

## Discussion

The development of a CVA10 vaccine has been greatly impeded by the low proliferation rate of most CVA10 clinical isolates in the standard Vero cell culture. To overcome this issue, many studies focus on the development of non-Vero cell platforms or the cloning of Vero-adapted CVA10 strains. It has been reported that KMB17 and HEK293A cells could successfully propagating the immunogenic CVA10 [11,26,34]. Vero cell is a standard cell line for the production of poliovirus and EV-A71 vaccines, and this platform is a well-established and economical production system for enteroviral vaccine production. In recent years, several Vero cell-adapted CVA10 strains have been established for inactivated CVA10 vaccine development [11,13,20,23–25,34]. However, the mechanism by which these CVA10s adapt to Vero cells remains unclear.

RD cells are highly susceptible to coxsackieviruses so that it has been widely used to screen isolates from clinal samples [35]. The CVA10-M2014 strain was isolated from clinical samples and has been passaged serially in RD cells. The RD-propagated CVA10 strain (CVA10-R) isolated in this study was unable to propagate in Vero cells and has the P1 gene sequences similar to the parent strain (CVA10-M2014). In a previous EV-A71 vaccine candidate study, the MRC-5 cells were first used to culture the virus isolated from clinical sample, which was then transferred to Vero cell cultures [36]. Recently, several EV-D68 strains were adapted to MRC-5 and Vero cells, and the Vero-adapted EV-D68 strains were isolated from the MRC-5-adapted EV-D68 strains by serial passaging in Vero cells [37]. Based on the change of host cell tropism and the quasispecies theory of RNA virus [27], the CVA10-M2014 strain was first propagated in HEK293A cells in this study for increasing the virus yield and diversity. The resulting virus were then used to infect Vero cells and the Vero cell-adapted CVA10 strain (CVA10-V) was successfully isolated by following plaque cloning. Our results show that CVA10 presented different cell tropisms after propagate the virus in HEK293A cells, and the Vero-adapted strains displayed different properties from the parental strain. Following the preparation of CVA10-V in Vero cell culture, the purified CVA10-V F-particle induced strong immune responses in mouse study (Fig 3). In contrast, the CVA10-V E-particle did not induce an effective neutralization response, which is similar to the results of the E-particle of other coxsackieviruses in in previous studies [12,26]. Furthermore, when the CVA10-V F-particle was mixed with EV-A71 bulk to form a bivalent vaccine, a good neutralization response against CVA10 and EV-A71 were observed (Fig 3). These results show that CVA10-V is a suitable vaccine candidate for multivalent HFMD vaccine development.

Infectious clone is an important material for studying the characteristics and pathogenesis of an RNA virus. Many CVA10 infectious clones have been constructed by various promoter systems and used for virus characteristic researches [15]. In this study, in order to determine the crucial sites of CVA10’s adaptation to Vero cells, the infectious clones of CVA10-R and CVA10-V strains were successfully constructed and easily recovered as the CVA10-Rc and CVA10-Vc viruses (Fig 4). After comparing the sequences of CVA10-R and CVA10-V, five amino acids were altered in the P1 structural region (Table 1). Ten recombinant infectious clones were constructed, each containing a single amino acid change of the CVA10-Rc and CVA10-Vc (Fig 5A and 5C). Most CVA10-Rc mutants, except CVA10-Rc-E705D, could not propagate in Vero cells, while most CVA10-Vc mutants, except CVA10-Vc-D705E, could replicate in Vero cells. These results shown that the P1-705 position is the most critical amino acid for CVA10-V to infect Vero cells. In addition, the five mutants in the P1 region did not significantly affect the immunogenicity of CVA10 (Fig 3).

In an EV-A71 study, one amino acid substitution (VP1–145) was reported as a switch to induce a conformational change and affect the binding ability with the receptor [38]. In a recent study, a single mutation in VP1–143 of Coxsackievirus A6 cause phenotypic changes and altered VP0 cleavage efficiency [39]. Currently, the P1-705 position of CVA10 locates on the DE loop of VP1 (VP1–141) and exposes on the surface of the virion. Interestingly, the CVA10 VP1–141 position and the EV-A71 VP1–145 position are found to locate in the similar region in a previous alignment [3]. Due to the lack of CVA10 neutralizing antibodies, the virus attachment assay was used to evaluate the effect of P1-705 position of CVA10. The CVA10-Rc-E705D and CVA10-Vc-D705E mutants were tested with the parental CVA10 (its ability is defined as 100%), and the results showed that conversion between D and E of the P1-705 affects the attachment ability of CVA10 to Vero cells (Fig 6).

**Fig 7:**
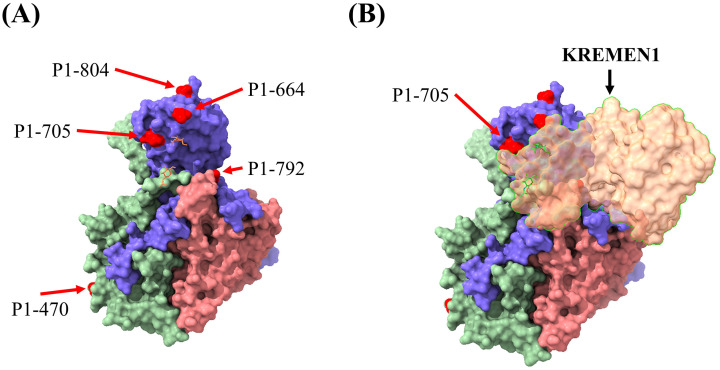
In silico modeling of CVA10-V mutation sites coupled with KREMEN1 receptor. **(A)** The location of five mutants of CVA10-V was labeled in the CVA10 structural model (PDB ID: 6SNW). **(B)** The KREMEN1 receptor was docked with CVA10 structural model.

In order to explore whether these mutation sites (P1-470, P1-664, P1-705, P1-792, and P1-804) interact with the KREMEN1 receptor, a structure of CVA10 complex with KREMEN1 receptor model (PDB ID: 6SNW) was constructed and analyzed by in silico simulation (Fig 7A; for detail analysis method, please refer to S1 File) [21]. The P1-804 position is located far from the KREMEN1 binding site. The P1-664, P1-705, P1-792 positions are located near the KREMEN1 binding site, while the P1-470 position is not. The KREMEN1 protein was overlayed onto the CVA10 structure, and the result showed that P1-705 site located at the edge of the interaction region of KREMEN1 and CVA10 VP1 (Fig 7B). Combining the results of the virus attachment and the simulation assays, it is possible that the VP1–141 (P1-705) position is an important site that contributes to the interaction between the KREMEN1 receptor and CVA10 when infecting Vero cells.

In a previous CVA10 study, human SCARB2 was found to be associated with the aiding of CVA10 infection [40]. We used ELISA to test the binding of CVA10-R and CVA10-V against human SCARB2 and PSGL-1 to see if specific binding affinity had altered between CVA10-R and CVA10-V (S3 Fig). We found that binding of SCARB2 with CVA10-R and CVA10-V did not differ significantly, which indicates SCARB2 interaction was not the source of the difference between CVA10-R and CVA10-V.

In conclusion, we isolated a Vero-adapted CVA10 (CVA10-V) strain through the two passages in HEK293A and then in Vero cells, which demonstrated to be successful in isolating a viable CVA10 clone for Vero cell culture. Inactivated CVA10-V F-particle elicited strong neutralization antibody responses against CVA10, and when mixed with an inactivated EV-A71 particle as a bivalent vaccine, good neutralization responses against CVA10 and EV-A71 could be observed (Fig 3). Specific CVA10-R and CVA10-V single-site mutants were constructed to determine the effects of variations of viral proteins on Vero infectivity. Our result indicates that the substitute of E to D at the P1-705 position is crucial for the CVA10-V to attach to Vero cells. This Vero-adapted CVA10-V strain would be beneficial for CVA10 vaccine production using the standard Vero cell culture system, but also aid in the development of multivalent HFMD vaccine.

## Supporting information

S1 File Supplement Materials and Methods. (DOCX)

S1 Table The primers for CVA10-R and CVA10-V. (DOCX)

S2 Table The primers for the construction of CVA10 mutant infectious clones. (DOCX)

S3 Table The primers for qPCR analysis. (DOCX)

S4 Table Nucleotide and amino acid residue changes in P1 of CVA10-V after 6 serial passages in Vero cells. (DOCX)

S5 Table Plaque sizes of CVA10 mutants. Three plaque morphology variants are shown: small (<1 mm); medium (1–2 mm); and large (>2 mm).
(DOCX)

S1 Fig Virus titer of serial passages of CVA10-V. CVA10-V virus has been passaged six generations, and the viral titer of each generation has been detected.
(DOCX)

S2 Fig Neutralization titers of mice sera against CVA6 and CVA16 viruses. The sera of mice immunized with CVA10-V E, CVA10-V F, and CVA10-V F + EV-A71 antigens, which was used to perform neutralization assay against CVA6 (strain M0746) and CVA16 (strain N5079) viruses respectively.
(DOCX)

S3 Fig Binding of CVA10-R and CVA10-V viruses against SCARB2 and PSGL receptors. Ninety-six well plates coated with live CVA10-R or CVA10-V (103 pfu) were incubated with various amounts of recombinant (A) hSCARB2-Fc or (B) hPSGL-1-Fc per well for 1 hour at room temperature. After incubation, the plates were washed three times with PBS, and then the CVA10 particles in the wells were quantified by incubating with GTX132346 antibody in an ELISA assay.
(DOCX)
