# Supplementary material for: VP1–141 is a determinant of a Vero cell-adapted Coxsackievirus A10 for vaccine development
Source: PLoS Negl Trop Dis. 2026 Jun 2;20(6):e0014396. doi: 10.1371/journal.pntd.0014396 (PMC13249402; doi:10.1371/journal.pntd.0014396)
Supplement: S1 File — (DOCX) [file pntd.0014396.s001.docx]

**Supplement Materials and Methods**

***M.1 Ethics statement***

The recombinant DNA application (NHRI-IBC-111346) was reviewed and approved by the Institutional Biosafety Committee of the National Health Research Institutes (NHRI), Taiwan. Animal experiments were conducted in accordance with the guidelines of the Laboratory Animal Center of NHRI, Taiwan. Protocols of animal study have been reviewed and approved by the NHRI Institutional Animal Care and Use Committee (Approved protocol No. NHRI-IACUC-110008-A and NHRI-IACUC-111114-A).

***M.2. Cells, media, and viruses***

The Vero cells (CCL-81) were obtained from the American Type Culture Collection (ATCC, USA). Rhabdomyosarcoma (RD) cells were obtained from the Bioresource Collection and Research Center (BCRC, Taiwan). Human embryonic kidney 293 adherent cells (HEK293A) were purchased from Invitrogen (Cat. R70507). Vero, RD, and HEK293A cells were cultured in Dulbecco's modified Eagle's medium (DMEM, Gibco) with 10% fetal bovine serum (FBS) for virus adaption. Vero cells were cultured in serum-free medium VP-SFM (Gibco) for virus propagation. The CVA10-M2014 strain was obtained from National Cheng Kung University Hospital, Taiwan. The EV-A71 E59 strain was obtained from the Centers for Disease Control, Taiwan.

***M.3. SDS-PAGE and western blotting analysis of viral proteins***

Western blotting analyses of samples were performed by 4-12% Bis-Tris SDS-PAGE (Invitrogen). The proteins were separated by SDS-PAGE and transferred onto a PVDF membrane (Invitrogen) using a Mini Trans-Blot Cell (Bio-Rad) according to the manufacturer’s instructions. The first antibodies were used to recognize the CVA10 antigen. The anti-CVA6 VP1 rabbit polyclonal antibody GTX132346 (GeneTex), which could recognize the VP1 of CVA6, CVA10, and CVA16, was used for detection [Lien et al., 2023]. The secondary, goat anti-rabbit IgG AP132P (Millipore) conjugated with horseradish peroxidase (HRP), was then applied Immobilon crescendo western HRP substrate WBLUR0500 (Millipore) was used for chemiluminescence development and detected by the Amersham Imager 600 system (GE Healthcare).

***M.4. The viral particles of CVA10-V* *preparation***

The Vero cells (2 x 10^7^ cells) were seeded into each CellBIND 850 cm^2^ roller bottle (Corning) containing 200 mL of culture medium in roller rack incubator at 37 ^o^C for cell propagation. After 6 days, the medium of each roller bottle was replaced with 333 mL fresh medium containing CVA10-V at the multiplicity of infection (MOI) = 0.0001. After 6 days post infection (DPI), the culture supernatant was harvested (total 1L) for virus purification. The CVA10 empty (E)-particle and full (F)-particle were isolated by continuous sucrose gradient ultracentrifugation. The cell debris was removed by passage through a 0.65 mm filter (Sartorius), and the supernatant was concentrated 20-fold with a 100 K TFF capsule (Sartorius). The crude CVA10-V concentrate was loaded onto a 10-50% continuous sucrose gradient and centrifuged at 32,000 rpm for three hours using a zonal rotor in a Hitachi CP80 ultracentrifuge. The fractions (50 mL per fraction) were collected and the infectivity of fraction was assessed by a TCID_50_ assay. The fractions were also subjected to SDS-PAGE and western blot analyses. The zonal centrifugation-purified CVA10-V virus was pooled and concentrated by diafiltration using an Amicon 100 K tube (Millipore) and centrifuged at 3,000 g, then stored at 4 ^o^C. The total protein concentration of the purified virus fractions was determined by a BCA protein assay.

***M.5. Transmission electron microscopy***

Transmission electron microscopy (TEM) was used to examine the purified viral particle samples. The purified CVA10 samples were inactivated by formalin solution (v/v 1:4000 dilution) at 37 °C for 3 days. The sample (4 µL) was loaded on carbon-vaporized copper grid (200-mesh) for 15 min at room temperature. The excess sample was removed with paper, and washing twice with water. The grid was stained with 2% uranyl acetate solution. The stained sample was air-dried and examined using the Joel JEM-1400 transmission electron microscope.

***M.6. Immunogenicity studies***

The purified, formalin-inactivated (v/v 1:4,000 dilution) samples of CVA10-V particles were adsorbed with aluminum phosphate. A group of 6 female BALB/c mice (6-8 weeks old) were immunized intramuscularly (i.m.) with 0.2 mL antigen (0.5 µg viral protein with 60 µg Alum) [1]. Formalin-inactivated EV-A71 bulk (sample-05), which was evaluated in our previous study [2], was used in the bivalent study. For bivalent test, EV-A71 bulk (0.5 μg) and CVA10-V F-particle (0.5 μg) were mixed with 60 µg aluminum phosphate in 0.2 mL. Mice were boosted twice with identical content at two-week intervals after priming. Blood from the immunized mice was collected one week after the final boost, and the serum was used for virus neutralization study.

***M.7. Virus neutralizing assay***

Serum samples collected from immunized mice were inactivated at 56 °C for 30 minutes. Each serum sample was then serially diluted (2-fold) with culture medium. Two hundred µL of virus solution with titer equal to 200 TCID_50_ were added to tubes containing 200 µL of the diluted sera. After incubation at 4°C for 18-24 hours, these samples (100 µL/well) were added to 96-well plates containing RD cells. The cultures were incubated for 6 days at 37 °C, and TCID_50_ were measured after quantifying the CPE in the infected RD cells. The Nt value is the geometric reciprocal of the serum dilution yielding a 50% reduction in the viral titer, was obtained using the Reed-Muench methods.

***M.8. Sequence alignment and three-dimensional homology modeling prediction***

The genome sequences of reported CVA10 strains were obtained from the NCBI PubMed website (http://www.ncbi.nlm.nih.gov/pubmed/ and aligned using the CLUSTALW program (https://www.genome.jp/tools-bin/clustalw). The three-dimensional structure of CVA10 (PDB: 6SNW) was used to predict the epitope position by homology modeling [3]. UCSF ChimeraX was used to display the position of the identified peptides [4].

**References**

1. Lien SC, Shen YS, Lin HY, Wu SR, Fang CY, Chen CH, Chen YA, Chong PC, Huang MH, Chow YH, Wang JR, Wu SC, Liu CC. Propagation and immunological characterization of coxsackievirus A10 in a serum-free HEK293A cell culture system. Virus Res. 2023;329:199101. doi: 10.1016/j.virusres.2023.199101.

2. Shen YS, Chow YH, Fang CY, Wu SR, Chen CH, Huang MH, Liao CL, Chiang JR, Liu CC. The stability and immunogenicity of formalin-inactivated Enterovirus A71 whole virion vaccine after ten years of low temperature storage. J Microbiol Immunol Infect. 2023;56(6):1121-1128. doi: 10.1016/j.jmii.2023.10.006.

3. Zhao Y, Zhou D, Ni T, Karia D, Kotecha A, Wang X, Rao Z, Jones EY, Fry EE, Ren J, Stuart DI. Hand-foot-and-mouth disease virus receptor KREMEN1 binds the canyon of Coxsackie Virus A10. 2020;11(1):38. doi: 10.1038/s41467-019-13936-2.

4. Meng EC, Goddard TD, Pettersen EF, Couch GS, Pearson ZJ, Morris JH, Ferrin TE. UCSF ChimeraX: Tools for structure building and analysis. 2023;32(11):e4792. doi: 10.1002/pro.4792.
