# Supplementary material for: VP1–141 is a determinant of a Vero cell-adapted Coxsackievirus A10 for vaccine development
Source: PLoS Negl Trop Dis. 2026 Jun 2;20(6):e0014396. doi: 10.1371/journal.pntd.0014396 (PMC13249402; doi:10.1371/journal.pntd.0014396)
Supplement: S1 Table — (DOCX) [file pntd.0014396.s002.docx]

**Supplementary Table 1. The primers for CVA10-R and CVA10-V.**

| **Fragment** | **Primer** | **Sequence (5’to 3’)** |
| --- | --- | --- |
| F1 | SalI_1F | ACCGTGTCGACTTAAAACAGCCTGTGGGTTGTA |
|  | MiuI_1R | CTGGTACGCGTCCAGTCTCTAGCCGGTGTG |
| F2 | MiuI_2F | ACTGGACGCGTACCAGCGCTACAGGC |
|  | SpeI_2R | GGGTTACTAGTGTAAGCTCCAAATTAACACCTTG |
| F3 | SpeI _3F | CTTACACTAGTAACCCTAGATACCAATGAGAAGT |
|  | NotI_3R | CTGGATCGCGGCCGCTTTTTTTTTTTTTTTTTTTTTTTTTTTTTTGCTATTCTGGTTATAACAAATTTACC |
