# Supplementary material for: VP1–141 is a determinant of a Vero cell-adapted Coxsackievirus A10 for vaccine development
Source: PLoS Negl Trop Dis. 2026 Jun 2;20(6):e0014396. doi: 10.1371/journal.pntd.0014396 (PMC13249402; doi:10.1371/journal.pntd.0014396)
Supplement: S2 Table — (DOCX) [file pntd.0014396.s003.docx]

**Supplementary Table 2. The primers for the construction of CVA10 mutant infectious clones.**

| **Mutation Site** | **Primer** | **Sequence (5’to 3’)** |
| --- | --- | --- |
| 470 (VP3 146) | 470A-f | GCCGCCATGCTGGGCACGC |
|  | 470T-f | ACCGCCATGCTGGGCACGC |
|  | 470-r | TTCTCTGTTGGCTGGTTGAGCG |
| 664 (VP1 100) | 664A-f | GCGGACACCACTGGGTATGCTACATGGG |
|  | 664T-f | ACGGACACCACTGGGTATGCTACATGGG |
|  | 664-r | TCCCCCATCTGTGAGGTTAACTACTCC |
| 705 (VP1 141) | 705D-f | GATAATGGAGAGGCTCGTCCGTACAT |
|  | 705E-f | GAGAATGGAGAGGCTCGTCCGTACAT |
|  | 705-r | AGTCGTTGTGACAAATGTGAATTCTGCG |
| 792 (VP1 228) | 792M-f | ATGATGGGCACTTTTGCAGTGAGAGTT |
|  | 792V-f | GTGATGGGCACTTTTGCAGTGAGAGTT |
|  | 792-r | ATTGTTTGGGCACAATCCGTATGTTGT |
| 804 (VP1 240) | 804K-f | AAGGCAAGTCAACTAAAACTACAGACTAGAGTGTACATG |
|  | 804R-f | AGGGCAAGTCAACTAAAACTACAGACTAGAGTGTACATG |
|  | 804-r | CCTACTAACAACTCTCACTGCAAAAGTGCC |
