# Supplementary material for: VP1–141 is a determinant of a Vero cell-adapted Coxsackievirus A10 for vaccine development
Source: PLoS Negl Trop Dis. 2026 Jun 2;20(6):e0014396. doi: 10.1371/journal.pntd.0014396 (PMC13249402; doi:10.1371/journal.pntd.0014396)
Supplement: S3 Table — (DOCX) [file pntd.0014396.s004.docx]

**Supplementary Table 3. The primers for qPCR analysis.**

| **Primer** | **Sequence (5’to 3’)** |
| --- | --- |
| A10q-F primer | TCCATGACGCTCTGGGAAAC |
| A10q-R primer | GTACGCGTCCAGTCTCTAGC |
