# Supplementary material for: VP1–141 is a determinant of a Vero cell-adapted Coxsackievirus A10 for vaccine development
Source: PLoS Negl Trop Dis. 2026 Jun 2;20(6):e0014396. doi: 10.1371/journal.pntd.0014396 (PMC13249402; doi:10.1371/journal.pntd.0014396)
Supplement: S4 Table — (DOCX) [file pntd.0014396.s005.docx]

**Supplementary Table 4. Nucleotide and amino acid residue changes in P1 of CVA10-V after 6 serial passages in Vero cells.**

| **Strain** | **Nucleotide**  **variation *** | **Amino acid**  **variation ^#^** | **Affected protein**  **(residue)** |
| --- | --- | --- | --- |
| CVA10-V-P6 | A2856G | T704A | VP1 |
|  | A2985G | N747D | VP1 |

*: The first letter indicates the nucleotide residue of parental virus; the middle number indicates the nucleotide location in genome; the last letter indicates the nucleotide residue of virus.

#: The first letter indicates the amino acid residue of parental virus; the middle number indicates the amino acid location in viral polyprotein; the last letter indicates the amino acid residue in virus.
