# Supplementary material for: VP1–141 is a determinant of a Vero cell-adapted Coxsackievirus A10 for vaccine development
Source: PLoS Negl Trop Dis. 2026 Jun 2;20(6):e0014396. doi: 10.1371/journal.pntd.0014396 (PMC13249402; doi:10.1371/journal.pntd.0014396)
Supplement: S5 Table — Three plaque morphology variants are shown: small (<1 mm); medium (1–2 mm); and large (>2 mm). (DOCX) [file pntd.0014396.s006.docx]

**Supplementary Table 5. Plaque sizes of CVA10 mutants.** Three plaque morphology variants are shown: small (<1 mm); medium (1 to 2 mm); and large (>2 mm).

| **Original derived strain** | **Small (<1mm)** | **Medium (1-2mm)** | **Large (>2mm)** |
| --- | --- | --- | --- |
| CVA10-M2014 | CVA10-V | CVA10-R  CVA10-Rc  CVA10-Vc | CVA10-M2014 |
| CVA10-R | CVA10-Rc-T664A  CVA10-Rc-E705D | CVA10-Rc-T470A | CVA10-Rc-V792M  CVA10-Rc-R804K |
| CVA10-V | CVA10-Vc-A470T  CVA10-Vc-A664T  CVA10-Vc-M792V  CVA10-Vc-K804R | CVA10-Vc-D705E |  |
