# Supplementary material for: VP1–141 is a determinant of a Vero cell-adapted Coxsackievirus A10 for vaccine development
Source: PLoS Negl Trop Dis. 2026 Jun 2;20(6):e0014396. doi: 10.1371/journal.pntd.0014396 (PMC13249402; doi:10.1371/journal.pntd.0014396)
Supplement: S1 Fig — CVA10-V virus has been passaged six generations, and the viral titer of each generation has been detected. (DOCX) [file pntd.0014396.s007.docx]

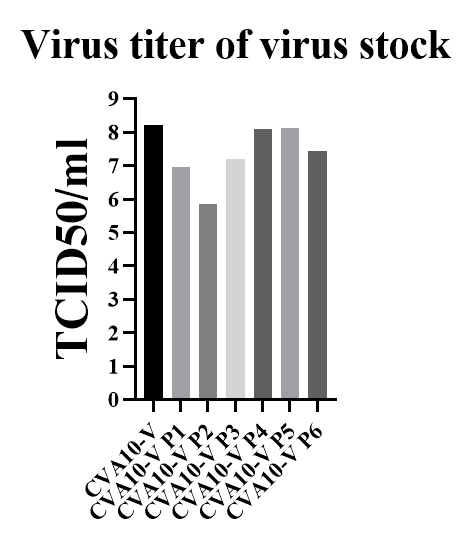


**Supplementary Figure S1. Virus titer of serial passages of CVA10-V.** CVA10-V virus has been passaged six generations, and the viral titer of each generation has been detected.
