# Supplementary material for: VP1–141 is a determinant of a Vero cell-adapted Coxsackievirus A10 for vaccine development
Source: PLoS Negl Trop Dis. 2026 Jun 2;20(6):e0014396. doi: 10.1371/journal.pntd.0014396 (PMC13249402; doi:10.1371/journal.pntd.0014396)
Supplement: S2 Fig — The sera of mice immunized with CVA10-V E, CVA10-V F, and CVA10-V F + EV-A71 antigens, which was used to perform neutralization assay against CVA6 (strain M0746) and CVA16 (strain N5079) viruses respectively. (DOCX) [file pntd.0014396.s008.docx]

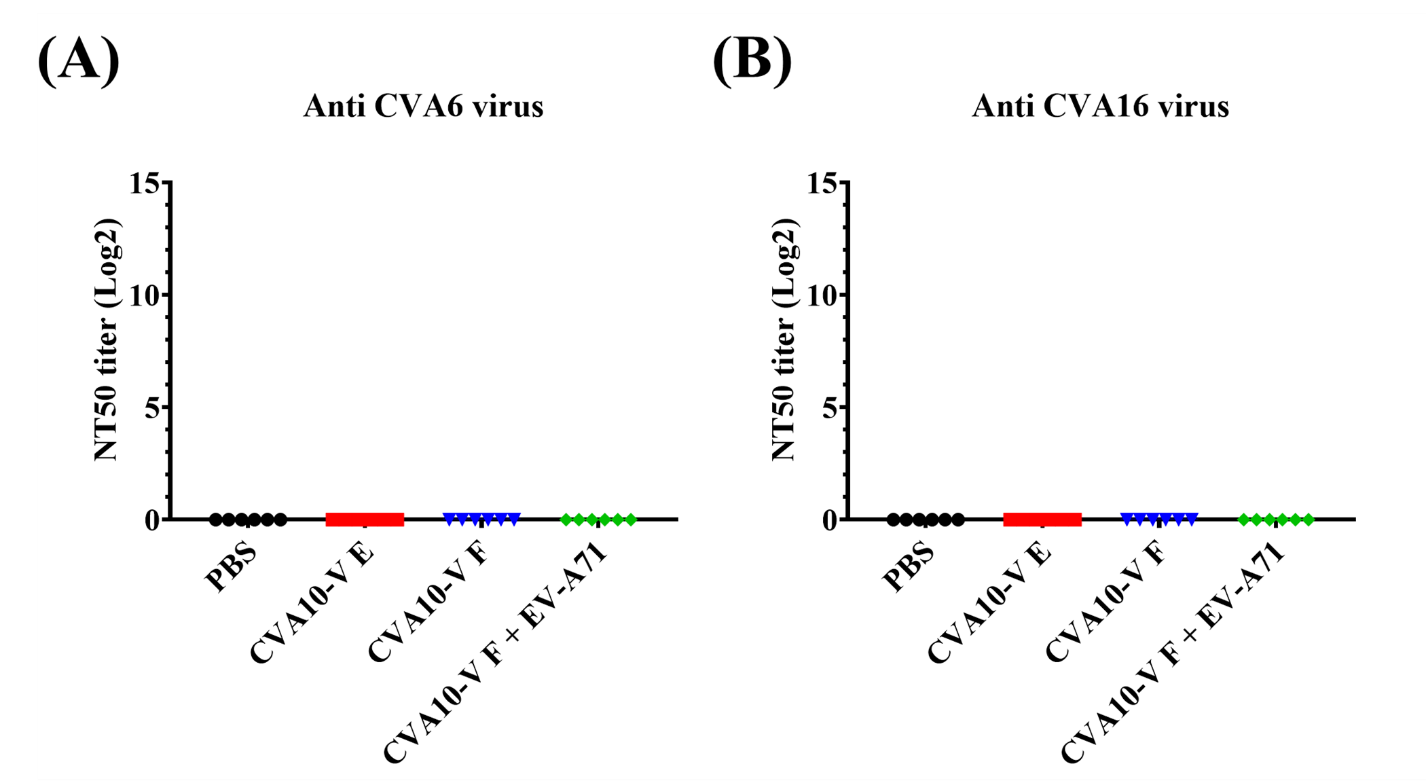


**Supplementary Figure S2. Neutralization titers of mice sera against CVA6 and CVA16 viruses.** The sera of mice immunized with CVA10-V E, CVA10-V F, and CVA10-V F + EV-A71 antigens, which was used to perform neutralization assay against CVA6 (strain M0746) and CVA16 (strain N5079) viruses respectively.
