# Supplementary material for: VP1–141 is a determinant of a Vero cell-adapted Coxsackievirus A10 for vaccine development
Source: PLoS Negl Trop Dis. 2026 Jun 2;20(6):e0014396. doi: 10.1371/journal.pntd.0014396 (PMC13249402; doi:10.1371/journal.pntd.0014396)
Supplement: S3 Fig — Ninety-six well plates coated with live CVA10-R or CVA10-V (103 pfu) were incubated with various amounts of recombinant (A) hSCARB2-Fc or (B) hPSGL-1-Fc per well for 1 hour at room temperature. After incubation, the plates were washed three times with PBS, and then the CVA10 particles in the wells were quantified by incubating with GTX132346 antibody in an ELISA assay. (DOCX) [file pntd.0014396.s009.docx]

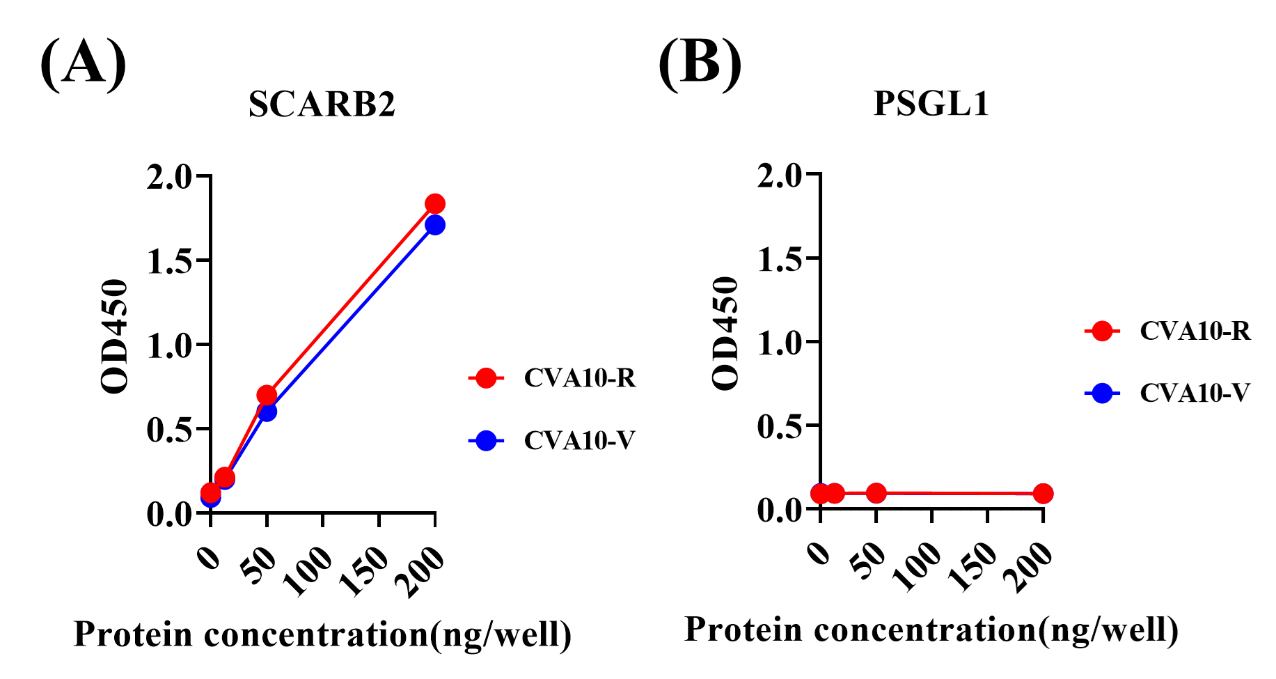


**Supplementary Figure S3. Binding of CVA10-R and CVA10-V viruses against SCARB2 and PSGL receptors.** Ninety-six well plates coated with live CVA10-R or CVA10-V (10^3^ pfu) were incubated with various amounts of recombinant (A) hSCARB2-Fc or (B) hPSGL-1-Fc per well for 1 hour at room temperature. After incubation, the plates were washed three times with PBS, and then the CVA10 particles in the wells were quantified by incubating with GTX132346 antibody in an ELISA assay.
